# Supplementary material for: Manic Fringe deficiency imposes Jagged1 addiction to intestinal tumor cells
Source: Nat Commun. 2018 Jul 31;9:2992. doi: 10.1038/s41467-018-05385-0 (PMC6068201; doi:10.1038/s41467-018-05385-0)
Supplement: Supplementary file 1 — Supplementary Information [file 41467_2018_5385_MOESM1_ESM.pdf]

Manic Fringe deficiency imposes Jagged1 addiction to intestinal tumor cells

López-Arribillaga et al.

Supplementary Figure 1

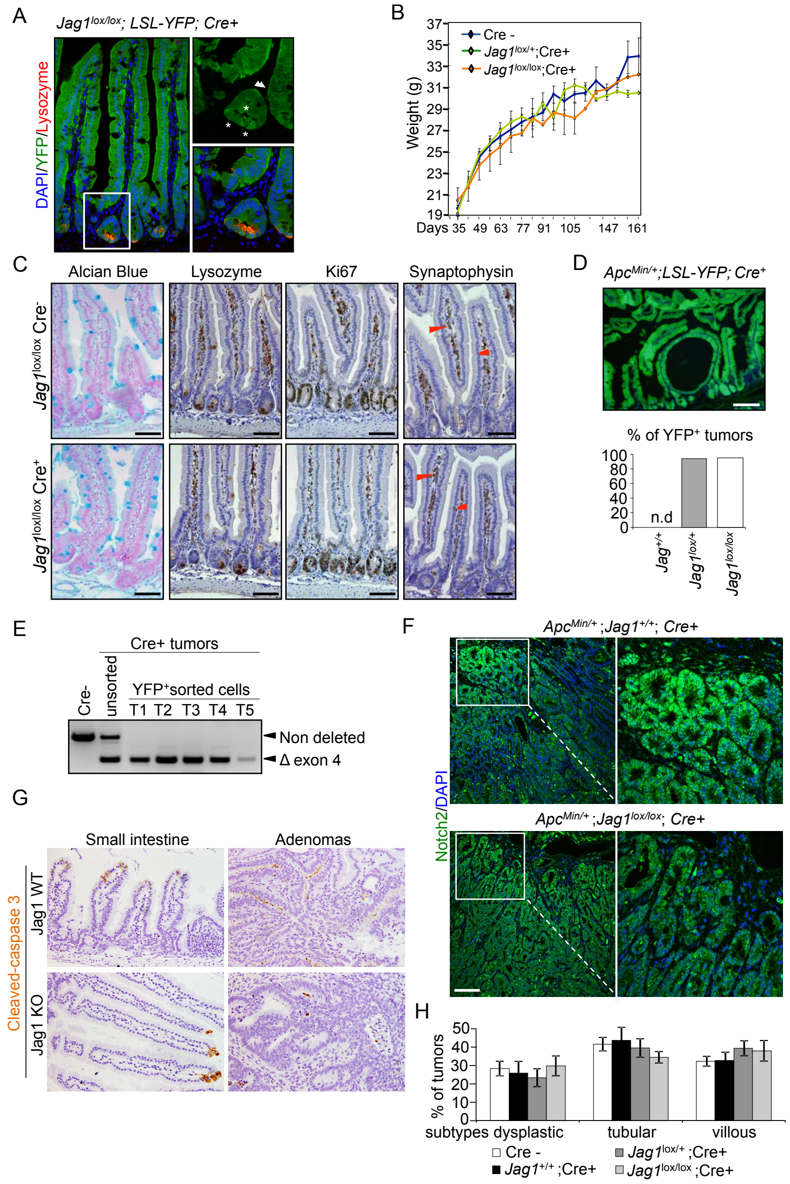

Intestinal-specific Jag1 deletion does not affect intestinal differentiation. (A) YFP expression in the small intestine of the mice indicates the distribution of CRE expression. YFP is shown in green whereas Paneth cells are labeled in red using α-lysozyme antibody. Nuclei are shown in blue (DAPI staining). Higher magnification images showing YFP^{+} ISCs located in between two Paneth cells (asterisk) and the +4 position (arrowhead). (B) Representation of mice weight determined weekly during six months. (C) IHC analysis of intestinal differentiation markers in the small intestine of the indicated mouse genotypes. (D) Quantification of the number of YFP-expressing tumors in the different mouse genotypes. (E) PCR analysis to determine the extent of Jag1-exon4 deletion in sorted cells (based on YFP expression) from different tumors (T1-5). (F) IF analysis of Notch2 expression in tumors from the indicated genotypes. A detail of the tumor area is shown in the right panels. (G) IHC analysis of cleaved-caspase 3 in the normal intestinal tissue and intestinal adenomas of Jag1 WT and Jag1 KO APCmin/+ mice. (H) Number of tumors with the indicated morphological features in the indicated genotypes (a minimum of 20 tumors per genotype were analyzed). In A, C, D and F scale bars represent 75 µm. In F right panels the scale bar is 25 µm.

Supplementary Figure 2

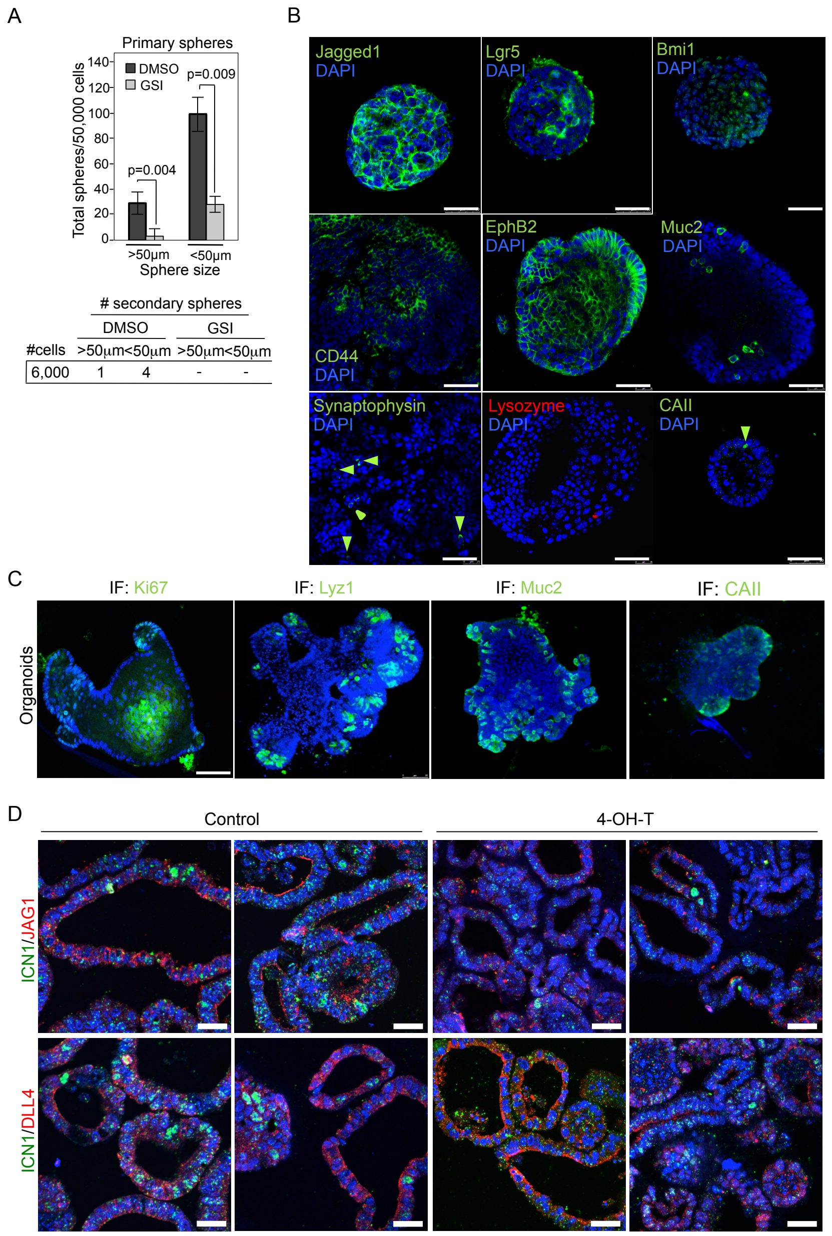

Spheroid growth is dependent on Notch activity. (A) Quantification of the number and size of the spheroids obtained in the control (0.13% efficiency in average) and the gamma-secretase inhibitor (GSI) treated cultures (70% of decrease in average). Secondary spheroids were generated after mechanical dissociation of primary spheres. (B-C) IF analysis of *Apc*$^{Min/+}$ intestinal spheroids (B) and wildtype organoids (C) using the indicated antibodies. (D) IF analysis of the indicated antibodies in *Apc*$^{Min/+}$ intestinal spheroids control or treated with 4-OH-T (5 µM, 36h) embedded in paraffin. Scale bars represent 50 µm in figure B and C, and 25 µm in D.

Supplementary Figure 3

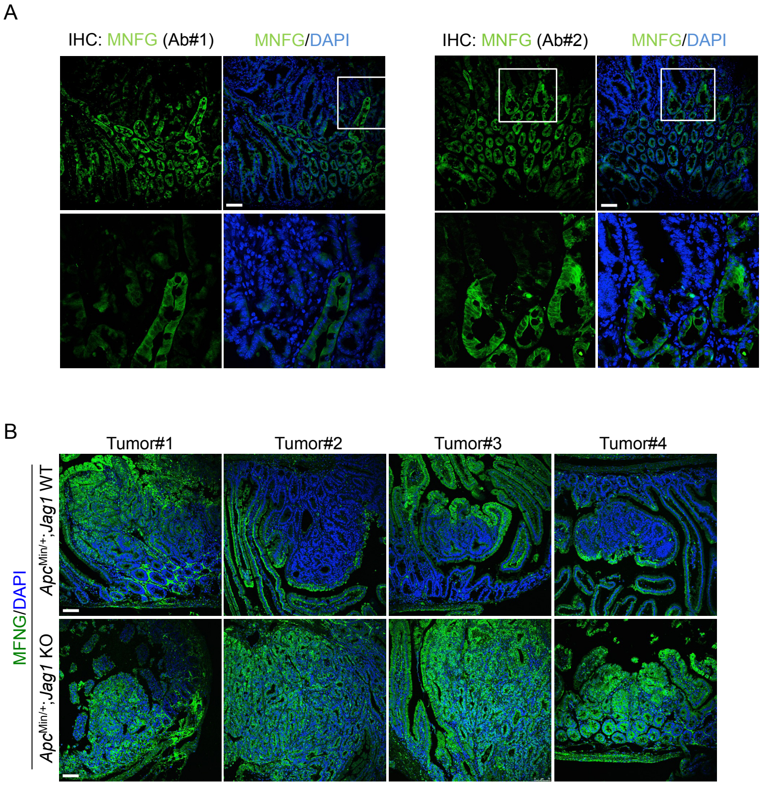

Manic Fringe is reduced in murine intestinal adenomas. (A) IF analysis of Manic Fringe (MNFG) expression in frozen sections of *Apc*$^{Min/+}$ intestinal adenomas using 2 different antibodies (see methods). (B) IF analysis of MFNG protein in 4 different adenoma samples from the indicated genotypes. Scale bars represent 75 μm in A and 50 μm in B.

Supplementary Figure 4

A

ADENOMA	ICN1 lo	ICN1 hi	TOTAL
JAG lo	28	34	**62**
JAG hi	15	58	**73**
**TOTAL**	**43**	**92**	**135**
Fisher's exact test	p=0.0015		
OR [95%CI]	3.18 [1.49 - 6.79]		

CARCINOMA	ICN1 lo	ICN1 hi	TOTAL
JAG1 lo	46	46	**92**
JAG1 hi	90	60	**150**
**TOTAL**	**136**	**106**	**242**
Fisher's exact test:	p=0.1431		
OR [95%CI]	0.68 [0.40-1.14]		

Breslow-Day test: p=0.0008

B

	MFNG negative	MFNG positive	TOTAL
**ADENOMA**	60	69	**129**
**CARCINOMA**	41	198	**239**
**TOTAL**	**101**	**267**	**368**
Fisher's exact test	p<0.0001		

C

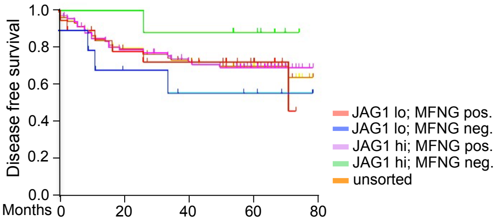

D

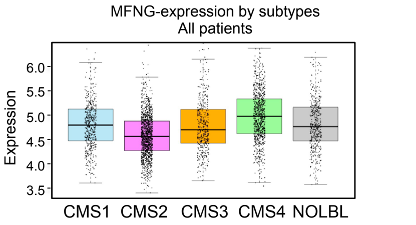

KW p-value = <2.22e-16

Pairwise MW-test fold-changes (p-values)

	Ref=CMS1	Ref=CMS2	Ref=CMS3	Ref=CMS4
CMS2	0.86 (<2.22e-16)			
CMS3	0.94 (0.18623)	1.09 (4.27e-10)		
CMS4	1.13 (1.221e-08)	1.31 (<2.22e-16)	1.20 (6.8232e-11)	
NOLBL	0.98(0.95884)	1.14 (3.2267e-15)	1.04 (0.24224)	0.87 (5.74e-08)

Impact of Manic Fringe expression levels on the regulation of specific pathways. (A) Table summarizing IF data for active ICN1 and JAG1 levels, and their correlation in a series of 135 adenoma and 242 carcinoma samples. (B) Table summarizing the IF data for Manic Fringe (MFNG) in the same samples. (C) Disease-free survival analyses (Kaplan Meier curves) of the indicated groups in the cohort of patients carrying ICN1 negative tumors. (D) MFNG expression by consensus molecular subtypes of colorectal cancer. Data include 2.822 colorectal cancer samples processed and submitted in the Synapse repository [1] by the Colorectal Cancer Subtyping Consortium [2]. Pairwise comparisons were performed using a Mann-Whitney test and expressed as fold-changes of group medians. A Kruskal-Wallis test was used for assess the global significance of the consensus classification.

Supplementary Figure 5

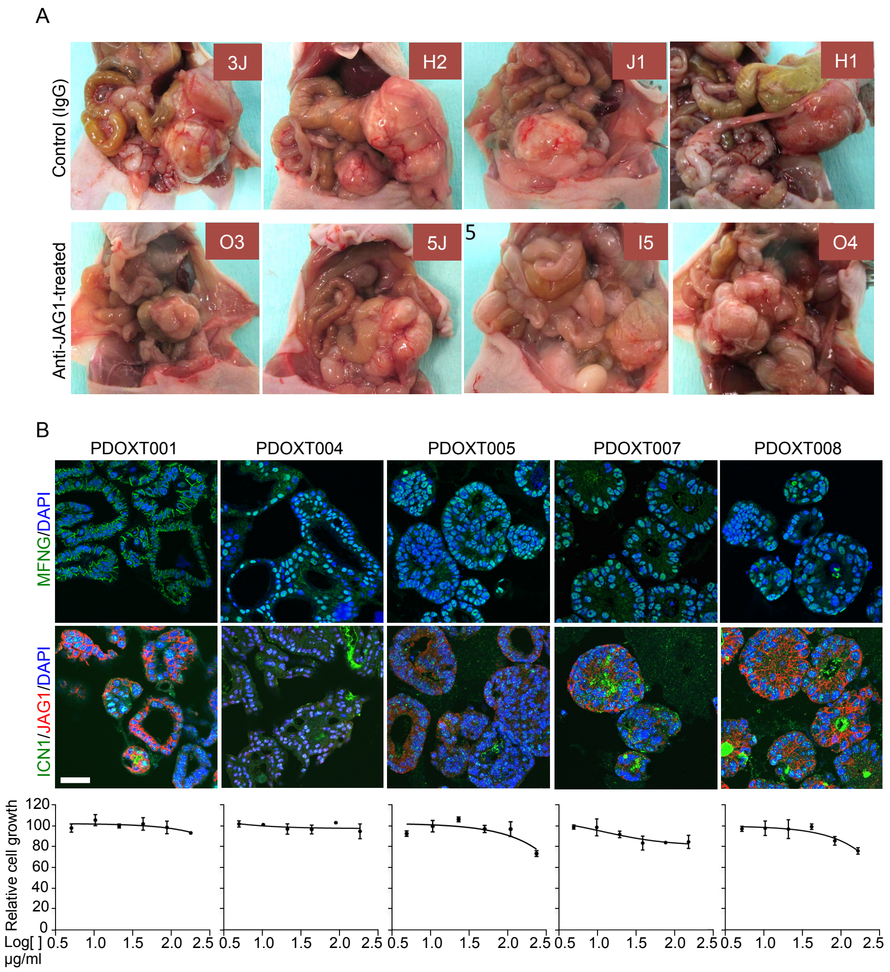

Anti-Jag1 antibody experiments in mice and patient-derived tumoroids. (A) Representative images of control and anti-Jag1 treated mice at the end of the experiment. (B) IF analysis of the indicated antibodies in different patient-derived colorectal cancer tumoroids and dose-response curves of the corresponding anti-Jag1 treated tumoroids.

Supplementary Table 1

List of Adenoma samples included in the analysis

Biopsy	Tumor Stage	ICN1	JAG1
07B7571-1P	Adenoma	high	low
07B9041-4	Adenoma	high	low
07B15509/1P	Adenoma	low	low
07B7219-1P1	Adenoma	high	low
06B12748-1XX	Adenoma	low	low
08B1837-1TBE3	Adenoma	low	low
08B10290/T6	Adenoma	low	low
07B1022-2XX	Adenoma	high	low
07B7068-1XX	Adenoma	high	low
07B5023-1P3	Adenoma	high	low
08B189-1P2	Adenoma	high	low
07B8812-3A	Adenoma	high	low
08B1837-1P2	Adenoma	low	low
07B10433-1P6	Adenoma	high	low
07B10433-1P7	Adenoma	high	low
07B9041-5	Adenoma	low	low
09B16124/3	Adenoma	high	low
10B4972/1	Adenoma	high	low
07B5023-1P1	Adenoma	high	low
07B5023-1P4	Adenoma	high	low
07B13648-3XX	Adenoma	high	low
10B4972/2A	Adenoma	high	low
07B5023-1P2	Adenoma	high	high
08B11407-2C	Adenoma	high	high
07B11905-1P4	Adenoma	high	high
08B1837-1P1	Adenoma	low	high
07B7219-1P5	Adenoma	low	high
07B7219-1P10	Adenoma	high	high
07B3080-1P1	Adenoma	high	high
07B12854-2XX	Adenoma	high	low
07B12854-3XX	Adenoma	high	low
07B10433-1P1	Adenoma	low	low
07B7219-1P8	Adenoma	low	low
07B7219-1P14	Adenoma	high	low

07B4145-1P2	Adenoma	high	low
09B10363/4	Adenoma	low	low
07B6418-2A	Adenoma	high	low
07B13224-1D	Adenoma	high	high
07B10433-1P8	Adenoma	high	high
07B13224-1C	Adenoma	high	high
07B5023-1P5	Adenoma	high	high
07B5023-1P8	Adenoma	high	high
08B792-1PO	Adenoma	high	high
07B7219-1P12	Adenoma	low	high
07B4077-P2	Adenoma	high	high
07B9041-8	Adenoma	high	high
09B16124/2	Adenoma	high	high
07B9242-3XX	Adenoma	high	high
10B7347/2	Adenoma	high	high
07B13224-1B	Adenoma	high	high
07B8812-1XX	Adenoma	high	high
07B7219-1P3	Adenoma	high	high
07B7219-1P7	Adenoma	high	high
08B820-3P	Adenoma	high	high
07B3332-1P	Adenoma	low	high
07B4368-3 P3	Adenoma	high	high
07B9330-1E	Adenoma	high	high
07B4965-1P2	Adenoma	high	high
07B1022-1XX	Adenoma	high	high
07B710-1XX	Adenoma	high	high
09B12849/1/P1	Adenoma	high	high
09B10363/3/B	Adenoma	low	high
07B1310-3	Adenoma	low	high
07B9330-1A	Adenoma	high	high
06B13949-1XX	Adenoma	high	high
07B5079/3	Adenoma	high	high
07B8635-P5	Adenoma	low	high
10B7347/6B	Adenoma	high	high
10B7347/12	Adenoma	high	high
07B3080-2XX	Adenoma	high	high
08B11407-2B	Adenoma	high	high
07B10433-1P3	Adenoma	high	high

07B7378-1XX	Adenoma	high	high
07B10378-P	Adenoma	high	high
07B6348/P1	Adenoma	low	high
09B5991-1P	Adenoma	high	high
07B7552-1	Adenoma	high	high
07B13648-1XX	Adenoma	high	high
08B11407-2A	Adenoma	high	high
07B9330-D	Adenoma	high	high
07B4368-3P1	Adenoma	high	high
07B9041-6	Adenoma	high	high
07B9330-C	Adenoma	high	high
07B14974/PO	Adenoma	high	high
07B4368-3P2	Adenoma	high	high
10B5105-1B	Adenoma	high	low
07B4475-1P	Adenoma	low	low
06B7163-1P	Adenoma	low	low
07B14625-1P1	Adenoma	high	low
06B11623-1P1	Adenoma	low	low
06B11623-1P2	Adenoma	high	high
07B5592-1P	Adenoma	high	low
07B15038	Adenoma	high	high
09B2781-1C	Adenoma	high	low
06B14020-P2	Adenoma	high	high
06B14020-P4	Adenoma	high	high
08B505-1	Adenoma	low	high
07B5023-1P7	Adenoma	high	low
07B13718-1P	Adenoma	high	high
07B6418-2B	Adenoma	high	high
07B11905-P1	Adenoma	high	high
09B10363/2	Adenoma	low	high
07B1310-2	Adenoma	high	high
10B7347/11	Adenoma	high	high
06B9813/P1	Adenoma	high	low
07B4965-1P1	Adenoma	low	low
06B13368-1P3	Adenoma	high	low
06B13770-2P	Adenoma	high	low
07B10633-2P	Adenoma	low	high
07B1306-2XX	Adenoma	low	low

10B7347/8	Adenoma	low	high
07B10633-2T	Adenoma	high	low
06B9813/P2	Adenoma	high	low
07B4145-1P1	Adenoma	high	low
08B820/2 P	Adenoma	low	high
06B6762-2XX	Adenoma	low	low
07B7219-1P11	Adenoma	high	low
10B9228/P1	Adenoma	high	high
10B9578/1	Adenoma	low	low
08B2239-4P	Adenoma	high	high
09B2781-1A	Adenoma	low	low
07B7720-1P2	Adenoma	high	low
08B10290/PE	Adenoma	low	low
06B6898-P	Adenoma	low	low
10B7347/5A	Adenoma	low	low
06B9558-1P	Adenoma	low	low
10B4972/2B	Adenoma	low	low
07B5023-1P6	Adenoma	low	low
10B7347/5C	Adenoma	low	low
06B11623-1P3	Adenoma	low	low
06B13954/2 P	Adenoma	low	low
07B3267-2XX	Adenoma	low	low
10B7347/10	Adenoma	low	low
07B7219-1P13	Adenoma	low	high
07B7720-1P1	Adenoma	low	high
09B2781-1B	Adenoma	n.d.	low
08B12334-3P	Adenoma	n.d.	high
066B5572/2	Adenoma	n.d.	high

Detection levels of Jagged1 (JAG1), active Notch1 (ICN1) in the IHC analyses are indicated. (n.d. = non determined).

Supplementary Table 2

Summary of IF stainings of carcinoma samples

**MFNG negative**	ICN1 lo	ICN1 hi	**TOTAL**
**JAG1 lo**	13	5	**18**
**JAG1 hi**	14	9	**23**
**TOTAL**	**27**	**14**	**41**
Fisher's exact test:	p=0.520		
OR [95%CI]	6.22 [0.63 - 62.16]		

**MFNG positive**	ICN1 lo	ICN1 hi	**TOTAL**
**JAG1 lo**	31	40	**71**
**JAG1 hi**	75	51	**128**
**TOTAL**	**106**	**91**	**197**
Fisher's exact test:	p=0.037		
OR [95%CI]	0.63 [0.31 – 1.30]		

Tables summarizing the IF data for Jag1 and ICN1 in the MFNG positive or negative carcinoma samples. Statistical significance of the correlations inside groups was determined by Fisher's exact test. Significance of the differences between MFNG positive and negative groups was determined by Breslow-day test.

Supplementary Table 3

List of carcinoma samples included in the analysis

Biopsy	Tumor Stage	ICN1	JAG1	DLL4	MFNG	N2
09B7739/T3	T3N0	high	high	high	pos	neg
09B3749/1K	T3N2M1	low	high	high	pos	neg
09B1248/3T8	T4N2	high	low	high	pos	neg
09B2368/T1	T3N1	high	low	high	pos	neg
09B2415/T1	T4N2	low	high	high	pos	neg
09B1709/T2	T2NO	high	high	high	pos	neg
09B4130/T5	T3N1	low	high	low	pos	neg
09B3800/T5	T3N1	low	high	high	pos	neg
09B5567/T6	T3N1	high	high	high	neg	neg
09B8805/T2	T?NO	low	high	high	pos	neg
09B9605/T3	T4N1	low	high	high	pos	neg
09B7139/T8	T3N2	high	high	high	pos	neg
09B2985/T4	T3N0	low	high	high	pos	pos
09B8464/R6	T2N2	high	high	high	pos	pos
09B3920/T15	T3NX	high	low	high	pos	neg
09B2317/R2	T3N0	low	high	high	pos	neg
09B5597/T6	T3N0	high	high	high	pos	neg
09B3275/T5	T3N0	high	high	high	pos	pos
09B3409/T4	T3N0	low	high	low	pos	neg
09B15136/T2H	T3N0	low	high	high	neg	neg
09B8553/2T4	T3N0	low	high	high	pos	neg
09B905/T7	T3N0	low	high	high	pos	neg
09B8504/T1	T3N0	low	high	high	pos	neg
09B4777/T9	T3N2	low	high	high	pos	neg
09B3043/T6	T3N0	low	low	high	neg	pos
09B8001/T11	T3N2	low	high	high	pos	pos
09B16397/T6	T3N0	high	high	high	pos	neg
09B11271/T12	T4N0	high	high	high	pos	neg
09B12326/3T4	T4N0	low	low	high	pos	neg
09B12997/T6	T4N0	low	high	low	pos	neg
09B11272/T5	T3N0	low	low	high	pos	neg
09B12620/T9	T4N0	low	low	low	pos	neg
09B11037/1E	T3N0	low	low	high	pos	neg
09B10708/T3	T3N0	low	low	high	pos	neg

09B10826/T2B	T3N1	low	high	high	pos	neg
09B16116/T3	T4N1	high	high	high	pos	pos
09B12988/4T4	T4N1	high	high	high	pos	neg
09B12327/T3	T3N0	low	high	high	pos	neg
09B12810/T2	T2N0	high	high	low	pos	neg
09B10940/T3	T2N1	low	high	high	pos	pos
09B10548/T1	T4N1	low	high	high	pos	pos
09B11514/T3	T4N1	high	high	high	pos	neg
09B11164/T2	T3N0	low	high	high	pos	neg
09B13937/T3	T3N1	high	high	high	pos	neg
09B11728/T6	T4N2	low	low	high	pos	neg
09B13561/T5	T3N0	low	high	high	pos	neg
09B16742/T6	T4N0	high	high	high	pos	pos
09B9717/T5	T3N2	low	low	high	pos	neg
09B15768/T11	T4N1	low	high	low	neg	neg
09B14967/T7	T4N1	high	high	high	pos	neg
09B16259/T2	T2N1	high	high	high	pos	pos
09B14843/T4	T3N2	low	high	high	pos	neg
09B13908/T4	T4N2	high	high	high	pos	neg
09B10186/T9	T3N0	low	high	high	pos	neg
09B4585/T6	T3N0	low	high	high	pos	neg
09B5500/T2	T3N1	high	high	high	pos	pos
09B3582/T8	T3N0	low	low	high	pos	neg
09B8818/4T10	T3N0	low	high	high	pos	neg
09B5800/T7	T3N0	low	high	high	pos	neg
09B5938/T1	T3N2	low	high	high	pos	neg
09B9538/T5	T3N0	low	high	high	pos	neg
09B8505/T4	T3N1	low	low	high	pos	neg
09B5154/4T5	T3N0	high	high	high	pos	pos
09B7630/T1	T3N1	low	high	high	pos	neg
09B8258/T10	T3N0	high	high	high	pos	pos
09B6319/T6	T4N2	high	high	high	pos	neg
09B10107/T1	T3N0	high	low	high	pos	neg
09B8377/T1	T3N2	low	low	high	pos	neg
09B16168/T2	T3N0	low	low	high	neg	neg
09B6630/T3	T3N0	low	low	high	pos	neg
09B16809/T7	T3N0	low	low	n.d.	pos	neg
09B12164/2T7	T4N0	low	low	n.d.	pos	neg

09B9903/T6	T3N1	high	high	n.d.	pos	neg
09B1462/T3	T3N1	low	low	n.d.	neg	neg
09B15170/2A	T4N1	high	low	n.d.	pos	neg
09B11704/T4	T3N0	high	high	n.d.	pos	neg
09B9434/T2	T3N2	high	high	n.d.	pos	pos
09B15867/T3	T4N0	high	high	n.d.	pos	pos
09B17300/T9	T4N1	high	high	n.d.	neg	pos
09B6512/2T1	T4N0	high	high	n.d.	pos	pos
09B11487/T2	T3N0	high	high	n.d.	pos	pos
09B15491/T9	T4N0	low	high	n.d.	pos	neg
09B17238/T7	T4N1	low	high	n.d.	pos	neg
09B13365/T4	T3N1	low	high	n.d.	pos	neg
09B5561/T5	T3N1	low	high	n.d.	pos	pos
09B6827/T8	T3N0	high	low	n.d.	pos	neg
09B13349/T4	T3N1	high	high	n.d.	pos	pos
09B8755/T2	T3N1	low	high	n.d.	neg	neg
09B16946/T2	T3N0	low	high	n.d.	pos	neg
10B3819/T9	T3N0	high	high	n.d.	pos	neg
09B16276/T2C	T3N0	low	high	n.d.	neg	pos
09B13296/L4C	T3N0	low	high	n.d.	pos	neg
09B14763/T6	T3N0	high	high	n.d.	pos	neg
09B16890/T1	T3N0	low	high	n.d.	pos	neg
09B12849/T4	T3N0	high	low	n.d.	pos	neg
10B5039/T5	T3N1	low	high	n.d.	pos	pos
10B3445/T9	T3N0	low	high	n.d.	pos	neg
10B495/T7	T3N0	low	high	n.d.	pos	neg
10B581/T3	T3N2	low	high	n.d.	pos	neg
10B154/T9	T4N1	high	low	n.d.	pos	neg
10B124/T6	T3N1	high	high	n.d.	pos	neg
10B933/T4	T3N1	low	high	n.d.	pos	pos
10B1383/T3	T3N0	low	high	high	pos	neg
10B4658/T6	T3N0	low	high	high	neg	pos
10B14075/T2	T3N0	high	high	high	neg	neg
10B8347/T2	T3N0	low	high	low	pos	neg
10B505/2T2	T3N0	low	high	high	pos	pos
10B8539/T5	T3N0	low	high	high	pos	neg
10B8414/T6	T3N0	high	high	low	pos	neg
10B9279/T5	T3N0	high	low	high	pos	neg

10B9228/T3	T3N0	high	high	high	neg	neg
10B8969/T5	T3N0	low	high	high	pos	pos
10B4230/1M	T2N1	low	high	high	neg	pos
10B9032/T4	T3N0	low	high	high	pos	neg
10B9465/T9	T3N0	low	high	low	pos	pos
10B1676/T8	T4N2	high	high	high	neg	neg
10B24407T4	T3N1	high	low	high	pos	neg
10B3818/K	T3N0	high	high	high	pos	neg
10B1378/T10	T3N1	low	high	low	neg	neg
10B1107/T1	T3N2	high	high	high	pos	neg
10B5401/T5	T4N0	high	high	low	neg	neg
10B10894/T3	T3N0	low	high	high	neg	neg
10B13934/T2	T4N0	high	high	high	pos	pos
10B12559/T6	T3N0	low	low	high	neg	pos
10B12738/T2	T3N0	high	high	low	pos	pos
10B13088/T3	T3N0	low	high	high	neg	neg
10B3010/T5	T2N0	low	low	low	pos	pos
10B10533/T4	T3N0	high	low	low	pos	neg
10B10457/T6	T3N0	low	high	high	pos	neg
10B9821/T4	T4N0	low	high	low	pos	pos
10B17167/2G	T1N0	low	low	low	neg	neg
10B17295/2B2	T2N0	high	high	high	pos	neg
10B2783/T7	T3N1	high	low	high	neg	pos
10B1692/T5	T4N2	low	low	high	neg	neg
10B12067/T3	T3N0	low	low	high	neg	pos
10B11294/T8	T3N0	low	low	low	neg	pos
10B9031/3T7	T4N2	low	high	n.d.	pos	neg
10B11877/T10	T4N1	high	high	n.d.	neg	neg
10B10683/T3	T4N1	low	high	n.d.	pos	neg
10B11463/T3	T3N1	high	high	n.d.	pos	pos
10B11550/T1	T1N1	low	low	n.d.	pos	neg
10B12889/T7	T3N2	low	high	n.d.	pos	pos
10B12532/T3	T4N1	low	high	n.d.	pos	neg
10B12411/1F1	T4N1	high	high	n.d.	pos	pos
10B13967/T1	T3N1	low	high	n.d.	pos	pos
10B13067/T7	T3N2	low	low	n.d.	pos	pos
10B12211/T3	T3N1	high	low	n.d.	pos	neg
10B15046/T3	T1N0	high	high	n.d.	pos	pos

10B14169/T1	T3N2	low	low	n.d.	pos	neg
10B2569/T3	T2N1	high	high	n.d.	pos	neg
10B16233/T6	T3N0	high	low	n.d.	pos	pos
10B16834/T6	T3N0	high	low	n.d.	pos	neg
10B15682/T5	T3N0	high	low	n.d.	pos	neg
10B9498/T6	T3N0	low	high	n.d.	pos	neg
10B11814/T2	T3N0	high	low	n.d.	pos	neg
10B2532/T1	T2N0	low	high	n.d.	pos	neg
10B15443/T1	T3N2	low	low	n.d.	pos	neg
10B10034/T6	T3N2	high	low	n.d.	pos	neg
10B10440/T2	T4N2	low	high	n.d.	pos	pos
10B7422/T7	T4N0	low	high	n.d.	pos	pos
10B14986/T3	T2N1	high	low	n.d.	pos	pos
10B17392/T7	T3N1	high	high	n.d.	pos	pos
10B7782/T4	T3N1	low	high	n.d.	pos	neg
10B9899/T5	T4N1	low	high	n.d.	pos	neg
10B11438/T2	T3N0	low	high	n.d.	pos	pos
10B5326/2T4	T4N1	low	high	n.d.	pos	neg
10B7492/T5	T3N1	low	high	high	pos	neg
10B5527/T1	T4N2	low	high	high	pos	neg
10B13153/T5	T3N0	high	high	high	pos	pos
10B12591/T6	T4N0	high	low	high	pos	neg
10B325/T2	T3N0	high	high	low	pos	pos
10B1842/T9	T3N0	low	high	high	pos	pos
10B10355/2T6	T3N2	low	low	high	pos	neg
11B2141/T1	T3N2	low	low	high	pos	neg
10B8133/T4	T4N0	low	high	high	pos	pos
11B3144/T10	T3N0	low	high	high	pos	neg
10B3081/T4	T3N1	low	high	high	pos	neg
10B17009/T4	T3N0	low	high	high	pos	neg
10B7228/T8	T3N0	high	high	high	pos	neg
10B5120/T3	T4N0	high	high	high	pos	neg
10B4240/T4	T4N0	high	low	high	pos	neg
11B1760/T5	T2N1	high	high	high	pos	neg
10B12289/T6	T3N0	high	low	high	pos	neg
11B981/TD1	T3N1	low	high	high	pos	pos
10B10355/T2	T3N0	high	high	low	pos	neg
10B6664/T1	T3N0	low	high	high	pos	pos

06B10977/1T	low	low	n.d.	n.d.	n.d.
06B11078-1T	low	high	n.d.	n.d.	n.d.
06B13368-1T	low	high	n.d.	n.d.	n.d.
07B14625-1A8	low	low	n.d.	n.d.	n.d.
08B2239-3T2	low	low	n.d.	n.d.	n.d.
07B12118-1A	low	low	n.d.	n.d.	n.d.
06B15088-1T	low	low	n.d.	n.d.	n.d.
07b13224/1T	low	high	n.d.	n.d.	n.d.
08B1837-1 TA2	low	high	n.d.	n.d.	n.d.
09B17135/1/T2	low	high	n.d.	n.d.	n.d.
06B15161/T	low	high	n.d.	n.d.	n.d.
07B13718-1T2	low	low	n.d.	n.d.	n.d.
07B9863-1T	low	high	n.d.	n.d.	n.d.
07B3332-1T	low	high	n.d.	n.d.	n.d.
07B5023-1T	high	low	n.d.	n.d.	n.d.
07B10633-2T	high	low	n.d.	n.d.	n.d.
06B13949-2XX (T)	high	low	n.d.	n.d.	n.d.
07B1712-1T1	high	low	n.d.	n.d.	n.d.
08B792-1P	high	low	n.d.	n.d.	n.d.
07B9150-1T	high	high	n.d.	n.d.	n.d.
06B14020-1T2	low	low	n.d.	n.d.	n.d.
07B3080-1T	high	low	n.d.	n.d.	n.d.
07B4145-1T	high	low	n.d.	n.d.	n.d.
07B7720-1P2	low	low	n.d.	n.d.	n.d.
07B11560-2A	high	high	n.d.	n.d.	n.d.
06B9501-1T2	high	low	n.d.	n.d.	n.d.
07B10433-1T2	high	low	n.d.	n.d.	n.d.
07B4475-1T	high	low	n.d.	n.d.	n.d.
07B4965-1T	low	low	n.d.	n.d.	n.d.
07B8635-1PC	low	low	n.d.	n.d.	n.d.
10B4972/7	low	low	n.d.	n.d.	n.d.
07B4820-1T1	high	low	n.d.	n.d.	n.d.
07B5592-1T1	low	low	n.d.	n.d.	n.d.
06B14117-1T	high	low	n.d.	n.d.	n.d.
07b9330/1/T3	high	low	n.d.	n.d.	n.d.
07B8635-1PG	low	low	n.d.	n.d.	n.d.
10B9228/T4	high	low	n.d.	n.d.	n.d.
07B6961-1T	high	high	n.d.	n.d.	n.d.

06B11623-1T	high	low	n.d.	n.d.	n.d.
07B14719-1A1	high	low	n.d.	n.d.	n.d.
07B4368-3T	high	low	n.d.	n.d.	n.d.
07B2154-1R	high	low	n.d.	n.d.	n.d.
08B189-1T2	low	high	n.d.	n.d.	n.d.
07B13648-2P	high	low	n.d.	n.d.	n.d.
07B13811/T	high	low	n.d.	n.d.	n.d.
07B12854-1XX	high	high	n.d.	n.d.	n.d.
07B12854-5XX	high	high	n.d.	n.d.	n.d.
07B12854-4XX	high	high	n.d.	n.d.	n.d.
07B7552-2XX	high	low	n.d.	n.d.	n.d.
07B7219-1P12	low	low	n.d.	n.d.	n.d.
07b4077/1T	low	low	n.d.	n.d.	n.d.
07B4145-2T	low	low	n.d.	n.d.	n.d.
09B12849/1/T2	low	low	n.d.	n.d.	n.d.
07B2371-1T	low	low	n.d.	n.d.	n.d.
10B9578/2A	high	low	n.d.	n.d.	n.d.
07B9330-T5	high	low	n.d.	n.d.	n.d.
07B14974/T5	low	n.d.	n.d.	n.d.	n.d.
06B6348/T1	high	low	n.d.	n.d.	n.d.

Tumor staging (T1-4), the presence/absence of infiltrate in the lymph nodes (N) or metastasis (M), and the relative levels of active Notch1 (ICN1), Jagged1 (JAG1), Delta 4 (DLL4), Manic Fringe (MFNG) and Notch2 (N2) in the IHC analysis are indicated when available. (n.d. = non determined; pos =positive, neg = negative). Samples positive for Notch or JAG1 but negative for MFNG are shown in grey. In tumors that were ICN1-high but JAG1-low, DLL4 and MFNG positivity is also indicated.

Supplementary Table 4

List of primers used in the study

Target	Sense (5'-3')	Antisense (5'-3')
β2m	CTGACCGGCCTGTATGCTAT	CAGTCTCAGTGGGGGTGAAT
Bmi1	CCAATGAAGACCGAGGAGAA	TTTCCGATCCAATCTGCTCT
c-Myc	GCTGGAGATGATGACCGAGT	AACCGCTCCACATACAGTCC
EphB2	TTCTCACCTCAGTTCGCCTCTG	CAAACCCCCGTCTGTTACATACG
Gapdh	TGTTCCTACCCCCAATGTGT	TGTGAGGGAGATGCTCAGTG
Hes1	CGGCATTCCAAGCTAGAGAAGG	GGTAGGTCATGGCGTTGATCTG
Hopx	GAGGACCAGGTGGAGATCCT	TCCGTAACAGATCTGCATTCC
Jag1-del	CTACATACAGCATCTACATGC	TCAGGCATGATAAACCCTAGC
Lgr5	CGTCTTGCTGGAAATGCTTTGAC	AAGGCGTAGTCTGCTATGTGGTG
Lrig1	CCAAAAGCTGCATGAGTTGA	GCACCACTGGTATCCTCGAT
mTert	AGGGTAAGCTGGTGGAGGTT	GATGCTCTGCTCGATGACAA
Nrarp	GTACCTTCCCGCCAACTACC	GCGGACGTAGAGACTTAGCC

Supplementary references

1. Derry JM, *et al.* Developing predictive molecular maps of human disease through community-based modeling. *Nature genetics* **44**, 127-130 (2012).

2. Guinney J, *et al.* The consensus molecular subtypes of colorectal cancer. *Nature medicine* **21**, 1350-1356 (2015).
